# Chronic kidney disease and severe mental illness: a scoping review

**DOI:** 10.1007/s40620-023-01599-8

**Published:** 2023-04-08

**Authors:** Claire Carswell, Clodagh Cogley, Kate Bramham, Joseph Chilcot, Helen Noble, Najma Siddiqi

**Affiliations:** 1 Department of Health Sciences, University of York, York, UK; 2 School of Nursing and Midwifery, Queen’s University Belfast, Belfast, Northern Ireland UK; 3 School of Psychology, University College Dublin, Dublin, Ireland; 4 King’s College Hospital NHS Trust, London, UK; 5 Institute of Psychiatry, Psychology and Neuroscience, King’s College London, London, UK; 6 Hull York Medical School, York, UK; 7 Bradford District Care NHS Foundation Trust, Bradford, UK

**Keywords:** Mental illness, Mental health, Health inequalities, Chronic kidney disease, Kidney failure

## Abstract

**Background:**

People who have severe mental illness experience higher rates of long-term conditions and die on average 15–20 years earlier than people who do not have severe mental illness, a phenomenon known as the mortality gap. Long-term conditions, such as diabetes, impact health outcomes for people who have severe mental illness, however there is limited recognition of the relationship between chronic kidney disease and severe mental illness. Therefore, the aim of this scoping review was to explore the available evidence on the relationship between chronic kidney disease and severe mental illness.

**Methods:**

Electronic databases, including MEDLINE, Embase, CINAHL, and PsycINFO were searched. The database searches were limited to articles published between January 2000–January 2022, due to significant progress that has been made in the detection, diagnosis and treatment of both SMI and CKD. Articles were eligible for inclusion if they explored the relationship between SMI and CKD (Stages 1–5) in terms of prevalence, risk factors, clinical outcomes, and access to treatment and services. Severe mental illness was defined as conditions that can present with psychosis, including schizophrenia, schizoaffective disorder, bipolar disorder, and other psychotic disorders. Thirty articles were included in the review.

**Results:**

The included studies illustrated that there is an increased risk of chronic kidney disease amongst people who have severe mental illness, compared to those who do not. However, people who have severe mental illness and chronic kidney disease are less likely to receive specialist nephrology care, are less likely to be evaluated for a transplant, and have higher rates of mortality.

**Conclusion:**

In conclusion, there is a dearth of literature in this area, but the available literature suggests there are significant health inequalities in kidney care amongst people who have severe mental illness. Further research is needed to understand the factors that contribute to this relationship, and to develop strategies to improve both clinical outcomes and access to kidney care.

**Graphical abstract:**

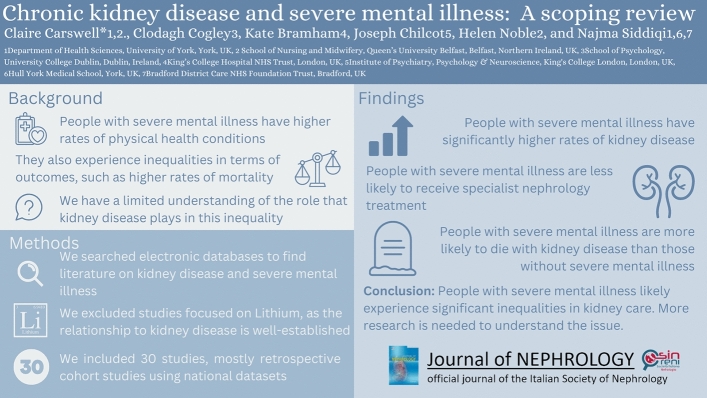

## Introduction

People with severe mental illness (SMI) experience significant health inequalities [1–3]. Severe mental illnesses are defined as mental health conditions that impair a person’s ability to function in their daily life, typically conditions that can present with psychosis, such as schizophrenia and bipolar disorder [3]. People who have an SMI diagnosis die, on average, 15–20 years earlier than people who do not [4]. One issue that contributes substantially to this inequality is the high prevalence of long-term conditions among people with SMI, and the poor clinical outcomes that people with coexisting SMI and long term conditions experience in comparison to those without SMI [3].

There has been ongoing research exploring the relationship between long term conditions and SMI [4], including developing interventions to help support people with SMI manage these long term conditions, for example diabetes mellitus [5] and cardiovascular disease [6]. However, some long term conditions have received relatively limited attention in research, despite the high prevalence of risk factors and the potential significant impact on clinical outcomes for people with SMI. One long term condition that is growing in prevalence, yet receives limited recognition, is chronic kidney disease (CKD) [7]. The most common causes of CKD in the general population are diabetes mellitus and hypertension, conditions that are significantly more prevalent among people with SMI, in part because of the side-effects of antipsychotic medication [2, 8, 9].

The early stages of CKD (stages I–III) can be difficult to detect due to lack of symptoms, yet early detection and intervention can help prevent progression of CKD, development of complications, and mortality. When kidney failure is reached people require kidney replacement therapy to sustain life. Kidney replacement therapy involves replacement of the damaged kidneys through either transplantation or dialysis. These options involve a significant treatment burden, with intensive monitoring and medication regimens, alongside regular invasive treatments, dietary restrictions, and fluid restrictions for dialysis treatment [10]. Research exploring self-management of long term conditions amongst people with SMI has highlighted the difficulties these individuals have managing conditions such as diabetes mellitus, cardiovascular disease, or long term conditions in general [11, 12]. However, kidney disease poses a unique and substantial burden in relation to self-management, for example haemodialysis may require people to attend hospital three times a week for 4 h each time or restrict fluid intake to only 500 mLs per day [10]. It is poorly understood how people with SMI experience this burden or engagement in the treatment of CKD or kidney failure [13].

Despite the increased risk of CKD associated with lithium [14], a common medication for bipolar disorder and schizoaffective disorder, the high prevalence of risk factors associated with the development of CKD, CKD progression and poor outcomes, we know very little about this co-morbidity, including prevalence, outcomes, and access to services. Therefore, this scoping review is needed to describe the available literature on this relationship and present what is already known about CKD and SMI.

### Aims

The aim of this review is to describe the available literature on the relationship between SMI and CKD, including prevalence, risk factors, clinical outcomes and access to treatment and specialist services.

## Materials and methods

A scoping review approach, following the steps outlined in the PRISMA-ScR reporting guidelines [15], was used due to the breadth of the review questions and the need to map the available evidence. A protocol for the scoping review was created to guide the searching, screening, data extraction and synthesis process, however as scoping review protocols are not eligible for registration on the PROSPERO database this was not registered.

### Information sources

Electronic databases, including MEDLINE, Embase, CINAHL, and PsycINFO were searched. The database searches were limited to articles published between January 2000–January 2022, due to significant progress that has been made in the detection, diagnosis and treatment of both SMI and CKD.

### Search strategy

A search strategy was developed for each of the two conditions (CKD and SMI) which were reviewed and revised by subject librarians. The two search strategies were combined using the Boolean Operator ‘AND’ to capture literature that explored the co-existence of both CKD and SMI. Table 1 provides an exemplar search strategy used in MEDLINE.

**Table 1:** MEDLINE Search strategy for CKD and SMI

Kidney disease search terms		Serious mental illness search terms
(renal transplant* or renal graft).tw. [48572] OR (kidney transplant* or kidney graft).tw. [45878] OR Nephrectomy/[35702] OR Kidney Transplantation/[100401] OR Kidney Diseases/[86554] OR Renal Insufficiency/[16761] OR kidney failure, chronic/[97374] OR (kidney failure or kidney disease).tw.[90202] OR chronic kidney disease.tw. [56082] OR Renal Dialysis/[96296] OR (hemodialysis or haemodialysis).tw.[81409] OR (end-stage kidney or end-stage renal or end stage kidney or end stage renal).tw. [45166] OR (ESKD or ESKF or ESRD or ESRF).tw. [19922] OR Renal Replacement Therapy/[6304] OR renal replacement therapy.tw [14286] OR dialysis.tw. [113773]	AND	(bipolar adj (disorder* or disease* or illness*)).tw,kf OR exp schizophrenia/(97,897) OR Affective disorders, psychotic/(2204) OR Bipolar disorder/(37,267) OR paranoid disorders/(3973) OR exp psychotic disorders/(48,180) OR schizo*.tw,kf. (119,094) OR (mani* adj3 depress*).tw,kf. (8267) OR (psychotic* adj3 depress*).tw,kf. (2369) OR (severe* adj3 affective*).tw,kf. (207) OR (severe* adj3 mental*).tw,kf. (9026) OR (severe* adj3 depress*).tw,kf. (8801) OR (psychos#s adj3 depress*).tw,kf. (3368) OR (serious* adj3 affective*).tw,kf. (39) OR "serious mood*".tw,kf. (24) OR (serious* adj3 mental*).tw,kf. (3957) OR (serious* adj3 depress*).tw,kf. (616)

### Eligibility criteria

Articles were eligible for inclusion if they explored the relationship between SMI and CKD (Stages 1–5) in terms of prevalence, risk factors, clinical outcomes, and access to treatment and services. Severe mental illness was defined as conditions that can present with psychosis, including schizophrenia, schizoaffective disorder, bipolar disorder, and other psychotic disorders.

During the initial scoping of the literature, it was apparent that there is an extensive body of literature establishing the relationship between lithium treatment and CKD, including recent systematic literature reviews and meta-analyses [16]. To avoid duplication, articles that focused on lithium either in terms of the mechanisms, prevalence, or risk factors for CKD, were excluded unless they provided insight into other contributing risk factors. As this review is focused on CKD, articles focusing on acute kidney injury were excluded. A more comprehensive list of exclusion criteria can be found in Table 2.

**Table 2:** Inclusion and exclusion criteria

Inclusion criteria	Exclusion criteria
Participants with a diagnosis of severe mental illness and chronic kidney disease (Stages 1–5), or participants who have experience providing care for people with severe mental illness and chronic kidney disease	Studies exploring the relationship between mental health and CKD, where mental health encompasses common mental disorders such as anxiety and depression
Articles published in the English language	Studies and articles focused exclusively on acute kidney injury
Empirical articles, including quantitative and qualitative studies	Studies and articles focused exclusively on the risk of CKD in lithium treatment
Participants over the age of 18	Participants under the age of 18
Articles focused on the relationship between SMI and CKD, including prevalence, risk factors, clinical outcomes, and access to services	Articles that are not reporting empirical research, for example editorial or news articles

### Data extraction

A data extraction table was created to capture the main components of the included articles and chart the available evidence. The data items that were extracted included author names and date of publication, publication type, setting, aim, study design, start date—end date, participants, relevant findings, and conclusions. Data were extracted by CC and CC, and were reviewed and discussed by all authors to confirm accuracy and relevance.

### Data synthesis

To address the aim of the scoping review, a descriptive narrative synthesis was carried out guided by the review question, providing an overview of the main areas of research to identify the current knowledge base and highlight gaps in the evidence. Due to the breadth of the review and heterogeneity of the included articles, no quantitative analysis was carried out.

## Results

**Fig. 1:**
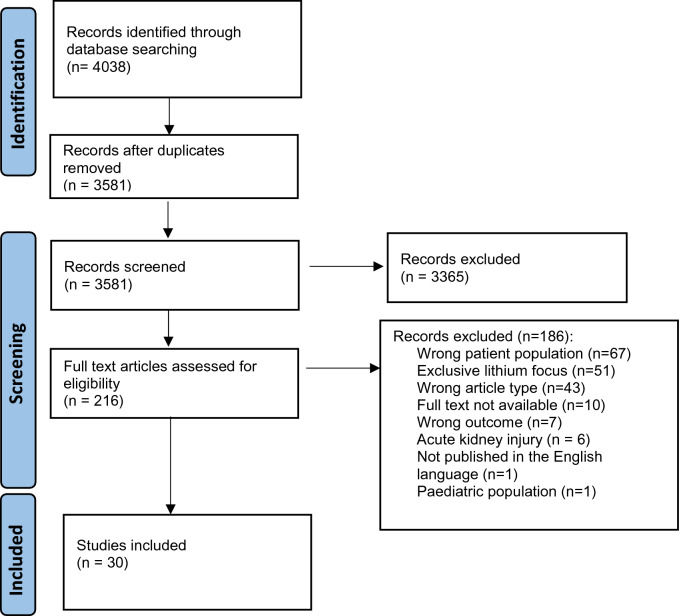
PRISMA flow diagram

A total of 3581 articles were identified from the database search once duplicates were removed. Following title and abstract screening, 3365 records were excluded due to their focus on topics that were irrelevant to the review, and 216 records were identified for full text screening. Following full text screening, 185 articles were excluded. Most articles were excluded because the studies focused on the wrong patient population (*n* = 67) or focused on the role of lithium in CKD (*n* = 51). An overview of the screening process is available in Fig. 1.

Thirty studies were identified for inclusion, reported in journal articles (*n* = 24) and conference abstracts (*n* = 6). Most included studies were retrospective cohort studies using national, regional, or local databases (*n* = 24), and cross-sectional analysis of healthcare databases (*n* = 5). One article reported a qualitative study. The studies were carried out in the USA (*n* = 11), UK (*n* = 5), Taiwan (*n* = 4), Denmark (*n* = 2), France (*n* = 2), Australia (*n* = 1), Canada (*n* = 1), India (*n* = 1), Ireland (*n* = 1), and Israel (*n* = 1). One study did not report the geographical location of study, although the authors were based in the UK.

An overview of the included studies is available in Table 3.

**Table 3:** Data extraction table

Study	Publication type	Setting	Aim	Study design	Start date—end date	Participants	Relevant findings	Conclusions
Alwar and Addis [45]	Journal article	UK, acute haemodialysis unit in a National Health Service Trust	To explore renal nurses’ experience of treating patients with SMI receiving acute haemodialysis to identify factors that facilitate or hinder nursing care of these patients	Qualitative study using semi-structured interviews	Not reported	*N* = 10 Renal nurses who had experience providing renal care to patients with SMI One senior nurse (Band 7) Four senior staff nurses (Band 6) Five staff nurses (Band 5)	Four main themes, nine subthemes: Perspectives of mental illness *Perception of mental illness* *Challenged by patient’s behaviour* *Attitude towards patients with mental illness* Patient and staff safety concerns: *Environment and staffing* Facilitators of care: *Respecting the patient* *Developing emotional resilience* *Understanding the behaviour behind the illness* Education and support needs: *Content and facilitation of education on mental illness* *Support for staff and patients*	Nurses experience individual challenges providing care to patients and managing patients with SMI in a haemodialysis setting Main barriers to providing effective care were staff shortages and lack of staff education Main facilitators to providing effective care were communication skills and empathy Improved support from, and collaborative working with, the mental health team is essential to improve quality of care
Chen et al. [28]	Journal article	Taiwan, National Health Insurance Database	To investigate sex-specific patterns of healthcare utilization and risks of psychiatric and physical comorbidities in the 3-month period before suicide mortality, in individuals with bipolar disorder	Nationwide retrospective cohort study / nested case control study	1/1/2000–31/12/2016	Patients who had a diagnosis of bipolar disorder (ICD codes 290–319) who did not have co-morbid schizophrenia, with at least one psychiatric hospitalisation for bipolar disorder Suicide cases = 1428 Living control patients = 5710	Significant differences between suicide cases and living controls in relation to employment and Charlson comorbidity index score. Patients who had died by suicide were more likely to have a higher comorbidity score CKD was more commonly present in the 3-month period pre-suicide in women, and only CKD and sepsis were associated with suicidality in male patients	CKD was more common in people with bipolar disorder, both men and women, who completed suicide in comparison to healthy controls
Boyle et al. [44]	Journal article	USA. Largest primary care clinic for patients with HIV Philadelphia, PA	To describe the characteristics of HIV-positive patients who were eligible for kidney transplant evaluation, and their rate of scheduled transplantation evaluation appointments	Retrospective cohort study	1/1/2008 – 31/12/2015	*N* = 42 Mean age: 47.4 (43.1–53.5) Male: 74% Black: 95% SMI prevalence: Schizophrenia: 14% (6) Bipolar disorder: 5% (2)	Patients who were not scheduled for kidney transplant evaluation appointments had higher prevalence of any psychiatric disorder and substance abuse There was a significant difference between prevalence of schizophrenia in those who did and did not receive a kidney transplant evaluation appointment	Only one person (out of 8) with an SMI diagnosis had a scheduled kidney transplant evaluation appointment
Garriga et al. [2]	Journal article	England, people registered with GPs	To describe NHS Health Check programme in people with mental illness-, rates of new diagnoses, and management of cardiovascular risk factors in those who attend NHS Health Checks, in comparison to people without mental-illness	Retrospective cohort study	April 2013 – March 2017	Patients were recorded as having SMI if they had a diagnosis of psychosis, schizophrenia, or bipolar disorder Total population eligible for checks = 3,492,816 People with SMI = 330,685 (9.5%) Total population attended: 590,218 People with SMI = 46,437 (7.9%)	People with SMI were more likely to be diagnosed with CKD at, or in the 12 months following, the health checks than people without SMI Adjusted HR: 1.23 (95% CI 1.12–1.34)	Antipsychotics and lithium may contribute to an increased risk of impaired kidney function in people with SMI, compared to those without SMI. Impaired glucose tolerance and metabolic syndrome are also likely to contribute to this increased risk
Germack et al. [36]	Journal article	USA, Medicare inpatient data for 5% national sample of beneficiaries	To analyse the hospital-level variation in unplanned readmissions for patients with SMI	Retrospective longitudinal cohort study	2013–2016	N (hospitals) = 2066 Individual participant demographics were not reported, instead hospital level demographics in relation to odds of increased readmissions were reported	People with SMI who were entitled to Medicare because of kidney failure were more at risk of readmission to hospital (OR = 2.025, 95% CI = 1.873 – 2.187)	Kidney failure is one of the strongest predictors for hospital readmission for patients who have SMI
Højlund et al. [25]	Conference abstract	Denmark, Funen Laboratory Cohort	To examine the association between use of second-generation antipsychotics (SGAs) and the risk of CKD	Population-based case–control cohort study	2001–2016	*N* = 21,434 incident CKD cases	Use of SGAs was associated with increased risk of CKD among ever-users (OR 1.24, 95% CI: 1.12–1.37) and current users (OR 1.26, 95% CI: 1.12–1.42) No clear evidence for a dose–response relationship Both short- and long-term prescriptions were associated with an increased risk of CKD Recent use of NSAIDs, prior use of lithium, hypertension, or prior AKI were not clearly associated with the development of CKD in connection to SGA exposure Highest risk of CKD was found in clozapine	Use of SGAs is associated with a small-to-moderately increased risk of incident CKD. All SGAs, except aripiprazole, were associated with an increased risk of CKD
Goswami Banerjee et al. [37]	Conference abstract	USA, Pennsylvania, Philadelphia	To evaluate risk factors for non-adherence in people with bipolar disorder who received a kidney transplant, compared to controls	Retrospective case–control study	1/3/2008 – 31/12/2016	*N* = 64 (BD = 32, Controls = 32) Mean age: 54 Female: 63% While: 88% Lithium-induced renal failure: 63% BD (0% controls)	**Patient survival (%):** 1 year post-transplant: BD = 96.88% Control = 100% 3 year post transplant: BD = 90.48% Control = 91.30% **Graft survival (%):** 1 year post-transplant: BD = 96.88% Control = 93.75% 3 year post-transplant: BD = 85.71% Control = 82.61%	The short-term outcomes for kidney transplantation are similar for people with bipolar disorder compared with matched controls
Kimmel et al. [32]	Journal article	USA, US Renal Data System	To assess the prevalence of hospitalisations with psychiatric diagnoses within a year of treatment initiation in adult and paediatric patients with ESRD and examine the associations between hospitalisations with psychiatric diagnoses and death in adult patients treated with dialysis	Retrospective cohort study	1996–2013	Patients with kidney failure enrolled in Medicare Part A and B (adults and children) = 1,033,958 Dialysis adult patients with hospital stays within 1 year observation period = 621,959 Dialysis adult patients followed from day 366 for all-cause death = 466,134	Psychiatric hospitalisations amongst people with kidney failure = Adult patients (22–64 yr) *N* = 108,309 Adult patients (> 65 yr) *N* = 142,402 SMI prevalence with psychiatric hospitalisation amongst people with kidney failure: Schizophrenic disorders as primary diagnoses: Adult patients (22–64 yr) *N* = 788 (1%) Adult patients (> 65 yr) *N* = 163 (0.1%) Other psychoses as primary diagnoses: Adult patients (22–64 yr) *N* = 418 (0.4%) Adult patients (> 65 yr) *N* = 694 (1%) The odds ratios of 1-year mortality were 1.23 (95% CI, 1.18–1.28) and 1.09 (95% CI, 1.08–1.11) in all adults hospitalised with primary and secondary psychiatric diagnoses, respectively, compared with those hospitalised without psychiatric diagnoses	Hospitalisations with psychiatric diagnoses are common in United States adult and paediatric patients on dialysis, and such hospitalisations are associated with higher mortality in adults In most hospitalisations psychiatric diagnoses were secondary (92% of hospitalisations
Manjunatha et al. [29]	Journal article	Community intervention studies in Bengaluru, in the southern state of Karnataka, and Turuvekere taluk, Tumakuru district, India	To understand the probable cause of death in patients suffering from schizophrenia in rural areas	Prospective cohort study	2009-NR	*N* = 584 patients enrolled in the interventions *N* = 55 patients who died during the follow-up time period Mean ± standard deviation age of deceased patients was 50.45 ± 13.65 years (25 patients were highest in the age range of 41–60 years) Almost equal gender distribution (male: female = 28:25)	Non-communicable diseases were the leading cause of death (*n* = 30) (followed by communicable disease) Among 25 people with recorded causes of death, 8 patients died due to CKD (2 of whom also had another underlying cause, TB, and COPD) (13.2%)	“NCDs are the most common causes of death among schizophrenia in two rural community cohorts. The high number of CKD in this study is a worrisome finding which needs further exploration.”
Tzur Bitan et al. [17]	Journal article	Israel, Clalit Health Services (CHS)	To assess the association between schizophrenia and CKD	Retrospective cohort study with matched controls	2000–October 2017	People with schizophrenia *N* = 27,516 Control *N* = 27,516 Male = 60.6%	In the cohort with schizophrenia, 1124 people (4.1%) had a diagnosis of CKD. In the control cohort, 671 people (2.4%) had a diagnosis of CKD Schizophrenia was found to be predictive of CKD above all other risk factors that were identified in the regression analysis (OR = 1.62, 95% CI = 1.45–1.82, *P* < 0.0001). CKD patients with no schizophrenia diagnosis were also significantly more likely to receive kidney transplantation treatment (5.9%) as compared to CKD patients with a schizophrenia diagnosis (1.6%), (OR = 5.43, 95% CI = 2.84–10.38, *P* < 0.001) Dialysis treatment was more commonly received among patients with CKD who did not have a comorbid schizophrenia diagnosis (11.1%) as compared to CKD patients who did have the diagnosis (8.5%)	Patients with schizophrenia are more likely to have comorbid CKD and are less likely to receive dialysis or kidney transplantation than patients without schizophrenia
Almeida et al. [18]	Journal article	Australia, Perth metropolitan region from Australian Electoral roll	To examine the cross-sectional and longitudinal associations between age of onset of bipolar disorder and clinical comorbidities, as well as incident dementia and mortality	A cross-sectional investigation of the association between diagnosis of older adults living with bipolar disorder and medical morbidities	1996–31/12/2013	*N* = 38,173 Australian men aged 65–85 Populations of interest were those who were diagnosed with bipolar disorder. Comparisons were made according to onset of bipolar disorder, with late onset bipolar disorder defined by two cut off points—50 years and 60 years	250 men had a diagnosis of bipolar disorder 170 had a recorded onset at or after 50 years Men with BD were as likely to have a diagnosis of cancer or renal disease as men without. (Renal disease was uncommon in the sample) Renal disease: OR (95% CI) BD onset > 50 years: 1.62 (0,38, 6.82) BD onset < 60 years: 1.36 (0.42, 4.39) BD onset > 60 years: 0.78 (0.11, 5.74)	The prevalence of renal disease is of particular concern in this age group in general, although renal problems were uncommon in the sample
Hamel et al. [38]	Conference abstract	USA, Pennsylvania, Philadelphia	To evaluate patient outcomes for people with bipolar disorder who received a kidney transplant	Retrospective cohort study	March 2008–December 2016	*N* = 29 Mean age = 55 ± 10 years Female = 69% White = 82.6% Lithium-induced renal failure = 66% Deceased donor = 83%	Biopsy confirmed rejection in one patient after 1 month. 2 patients received steroid pulses for presumed rejection within 6 months. No additional rejection episodes were recorded within the first-year post-transplant Patient survival (%): 1 year: 92.59 3 year: 94.12 Graft survival 1 year: 92.59 3 year: 94.12	Short-term outcomes for people who have bipolar disorder and receive a kidney transplant are similar to national data and were highly successful in this single centre
Iwagami et al. [19]	Journal article	United Kingdom, CPRD	Investigate the burden of CKD amongst people with SMI	Cross-sectional study	March 31st 2013-March 31st 2014	People aged 25–74 CKD population defined by eGFR < 60 mL/min/73m3 over at least 3 months Patients without SMI diagnosis (and no lithium use) = 2,387,988 Patients with SMI and no history of lithium use in CPRD = 24,101 Patients with history of lithium use in CPRD = 4295	Prevalence of CKD: 14.64% in people with SMI and a history of lithium prescription 3.34% in patients with SMI and no history of lithium prescription 2.09% in patients without SMI (*P* < 0.001) Evidence that there was higher renal replacement therapy for people with SMI (0.23% and 0.15%) with and without lithium history compared to those without SMI (0.11%) Different KRT modalities: Patients with SMI: 50% haemodialysis 2% Peritoneal dialysis 48% Kidney transplant Patients without SMI: 26% haemodialysis 7% peritoneal dialysis 66% kidney transplant The age–sex-adjusted odds ratio (OR) for CKD was 7.13 (95% confidence interval [CI] 6.47–7.85) for patients with SMI and history of lithium use, and 1.69 (1.56–1.83) for those with SMI and no history of lithium use, compared to those without SMI	There is a 6.5-fold increase in risk of developing CKD for people with SMI and a history of lithium prescription, and a 1.5-fold increase in risk for people with SMI and no history of lithium increase CKD is strongly associated with mortality and cardiovascular risk, so the increased risk of CKD could contribute to the mortality gap There was an increase in prevalence of KRT among people with SMI, with lower kidney transplantation rates, therefore it is important to identify whether inappropriate barriers exist to this treatment modality for people with SMI
Jackson et al. [34]	Conference abstract	USA, United States Renal Data System	To explore the specific risk factors for mortality in patients with schizophrenia and kidney failure	Retrospective cohort study	2005–2010	*N* = 2451 Age: 52 ± 9 years White: 50%	Significantly increased risk for death: Age > 35 (1.48 for 36–50 years, 1.87 for51–65 years) Catheter access vs AVF (1.34) Heart failure (1.17) Significantly decreased risk for death: Hypertension (0.33) Suicidal ideation (0.72) Drug dependence (0.84) Ischaemic cardiovascular (CV) disease (0.86) Non-compliance with treatment (0.77)	People with schizophrenia experience the same risk factors for mortality as people with ESRD without schizophrenia The factors associated with decreased risk of death are associated with escalations in care and close monitoring and follow up, which may explain this relationship
Kofman et al. [39]	Journal article	France, transplant departments	To compare the patient and allograft survival of patients with bipolar disorder who received a kidney transplant, with that of the general transplant population, and the outcome of psychiatric disease after transplant	Retrospective cohort study	1979–2014	Kidney transplant recipients with bipolar disorder or other psychotic disorder = 47 Women = 25 Men = 22 Patients with bipolar disorder = 34 (72%) Patients with other psychotic disorders = 13 (28%) These patients were compared with 94 controls matched for age, sex, and time of transplant	There are no significant differences between mortality and graft lost between people with SMI and people without SMI who received kidney transplants Post-transplant complications, such as infection and acute rejection episodes, were comparable to another retrospective study looking at transplant outcomes in patients with AA amyloidosis 23 patients had a relapse of their psychiatric disorder within 60 months follow-up. 13 people required hospitalisation in a psychiatric unit	There are similar mortality rates and graft loss rates in people with SMI compared to people without SMI, suggesting that SMI should not be an exclusion criterion for transplant Psychiatric conditions appear to be exacerbated in the early post-transplant period
Molnar et al. [40]	Journal article	United States, United States Renal Data System	To investigate the association of history of pre-transplantation psychosis/mania with post-transplant all-cause mortality and death with functioning graft, graft loss, rejection, and medication adherence	Retrospective cohort study	1/10/2018 – 31/3/2014	*N* = 122 propensity score matched transplant recipients with diagnosis of psychotic disorder *N* = 320 propensity score matched transplant recipients without diagnosis of psychotic disorder People with diagnosis of psychotic disorder: Mean age at baseline = 59 ± 9 years 90% male 60% White	Death with functioning graft: Crude mortality rate was similar in people with SMI (10 (8%) deaths, 37 per1000 patient-years, 95% CI: 20–70) versus people without SMI (29 (9%) deaths, 38 per1000 patient-years, 95% CI: 26–54) All-cause death: Compared to patients without history of SMI, patients with history of psychosis/mania had similar all-cause mortality risk (HR (95% CI): 1.04 (0.51–2.14)) Graft loss: The crude graft loss rate was similar in patients with history of psychosis/mania (14 (11%) graft loss, 52 per1000 patient-years, 95% CI: 31–88) versus patients without history of psychosis/mania (42 (13%) graft loss, 55 per 1000 patient-years, 95% CI: 40–74) Risk of Rejection Compared to patients without SMI, patients with history of SMI had similar risk of rejection (odds ratio (OR) (95% CI): 1.23 (0.60–2.53))	Recipients with history of psychosis/mania have similar survival, graft loss, and rejection risk compared to recipients without these diagnoses. This study suggests that transplantation can be safe even in patients with SMI
Wang et al. [26]	Journal article	Taiwan, National Health Insurance Research Database	To compare the risk of CKD between patients with schizophrenia using first- and second-generation antipsychotics	Population based nested case–control study	2000–2013	People with schizophrenia *N* = 3411 Controls *N* = 10,233 Male = 54.9% Mean age = 41.1 ± 10.2	More than 85% of subjects received both FGA and SGA medications. Taking patients using FGA only as reference group, the adjusted ORs (95%CI) for those who used no FGA and no SGA, SGA alone, both FGA and SGA were 0.53 (0.13–2.21), 1.06 (0.65–1.74), 1.28 (1.11–1.47), respectively (Table2). Patients that used both FGA and SGA have significant greater risk than patients used FGA only (*P* = 0.0009) Patients cumulatively used SGA 90–180 days and more than 1000 days have 42% and 30% significantly higher odds of developing CKD compared with the reference group (adjusted OR (95% CI) = 1.42 (1.06–1.91), 1.30 (1.13–1.51))	The risks for CKD for those who used SGAs longer cumulatively were higher than those who did not. In addition, those who used only FGAs and those who used both SGAs and FGAs seemed to have lower risk for CKD
Butler et al. [41]	Journal article	Ireland, Irish National Renal Transplant Programme	To compare outcomes, primarily in relation to patient survival, graft survival, and graft function, in patients with bipolar disorder and schizophrenia who undergo renal transplantation with renal transplant patients who do not have these diagnoses	Retrospective cohort study	1/1/1986 – 31/12/2013	*N* = 3000 Transplant recipients with bipolar disorder: 15 (0.5%) Transplant recipients with schizophrenia: 6 (0.2%) Patients with bipolar disorder: Age: 54 Male: 46.7% *Causes of kidney failure:* Chronic interstitial nephritis = 80% Hypertension *N* = 6.7% Renovascular disease = 6.7% Adult polycystic kidney disease = 6.7% Patients with schizophrenia: Mean age: 35 Male: 66.7% *Causes of kidney failure:* Chronic pyelonephritis = 33.3% Glomerulonephritis = 50% Renovascular disease = 16.7%	Across the three groups (general transplant recipients, recipients with bipolar disorder, and recipients with schizophrenia) there were no significant differences in graft survival over the follow-up time period, no significant difference in patient survival, no significant difference in graft function as measured by creatinine levels at 1 and 5 year post-transplant, no significant difference in the length of hospital admission post-transplant and no significant difference in frequency of acute rejection episodes	There is no evidence that SMI has a negative impact on transplant outcomes, and consequently patients with SMI should not be discriminated against or refused access to kidney transplantation, on the basis of mental illness alone
Hsu et al. [31]	Journal article	Taiwan, National Health Insurance Research Database	To investigate the prevalence of ESRD and mortality in patients with schizophrenia, and to assess the quality of pre-dialysis renal care and prognosis in dialysis-dependent ESRD patients with schizophrenia	Retrospective nationwide cohort study	2000–2014 (?) Stated to be a 14-year cohort study but no precise dates reported	Two different cohorts Matched Control (*n* = 54,361) vs ESRD-free schizophrenia (*n* = 54,361): Mean age: 41.1 Male: 54.8% DM comorbidity: 2.9% vs 4.3% Hypertension: 4.5% vs 4.8% Dialysis (*n* = 933) vs dialysis-schizophrenia (311): Age: 55 vs 54.9 Male: 42.1%	After 5 years of follow-up, there were 2,961 deaths and 143 ESRD subjects who required dialysis identified in the control subgroup, and 3,700 deaths and 85 ESRD subjects who required dialysis in the ESRD-free schizophrenia subgroup Lower risk of dialysis commencement amongst people with schizophrenia, but higher risk of death People with schizophrenia had a lower chance of seeing a nephrologist (OR = 0.6, 95% CI 0.4–0.8, P < .001), lower likelihood of receiving an EPO prescription (OR = 0.7, 95% CI 0.6–0.9, *P* <0 .05) and were more likely to be hospitalised within the first year after commencing dialysis (OR = 1.4, 95% CI 1.0–1.8, *P* <0.05)	People with schizophrenia were less likely to develop ESRD but had a higher risk of death compared to age matched controls. People with schizophrenia who did develop ESRD and received dialysis were less likely to receive adequate nephrology care A competing risk of death may explain the lack of progression to ESRD, or the lack of access and adherence to support may act as potential factors for increased mortality in people with SMI
Kessing et al. [27]	Journal article	Denmark, Danish population-based registers: Register of Medicinal Product Statistics14was linked with the Danish Medical Register on Vital Statistics, the Danish National Hospital Register, the Danish Psychiatric Central Register, and the Danish National Register on Regular Dialysis and Transplantation	To compare rates of CKD and end-stage kidney disease (defined as chronic dialysis or transplantation) among individuals exposed to lithium, individuals exposed to anticonvulsants and other drugs used to treat bipolar disorder and individuals not exposed to these drugs	Retrospective cohort study	1/1/1994–31/12/2012	Cohort 1 = 1,800,591 (all individuals) Definite CKD = 14,727 Cohort 2 = 10,591 (individuals with bipolar disorder)	Cohort 1 Trend tests confirmed statistically significant associations between the number of prescriptions of lithium and the rate of definite CKD (0 prescriptions: HR = 1.09 [95% CI, 0.81–1.45]; ≥ 60 prescriptions: HR = 3.65 [95% CI, 2.64–5.05]; *P* for trend < .001) and possible CKD but not for end-stage CKD Cohort 2 Rates of definite CKD and possible CKD did increase with: No. of prescriptions of lithium 1–2 prescriptions: HR = 0.89 [95% CI, 0.39–2.06] ≥ 60 prescriptions: HR = 2.54 [95% CI, 1.81–3.57]; *P* for trend < 0.001 No. of prescriptions of anticonvulsants 1–2 prescriptions: HR = 1.23 [95% CI, 0.76–1.99] ≥ 60 prescriptions: HR = 2.30 [95% CI, 1.53–3.44]; *P* for trend < 0.001 All trend tests showed statistically significant associations The rates of end-stage CKD did not increase with the number of lithium prescriptions whereas they significantly increased with the number of prescriptions of anticonvulsants (*P* for trend = 0.002)	The results suggest that CKD is associated with bipolar disorder, independent of medication. Both lithium and anticonvulsant treatment are associated with an increased risk of CKD, with anticonvulsant treatment being associated with kidney failure
Reilly et al. [20]	Journal article	UK, Clinical Practice Research Database	To examine the prevalence of SMI in the UK by country (England, Northern Ireland, Scotland and Wales) and region in England, and prevalence of 16 comorbidities in people with SMI	Retrospective cohort study	Three time periods: (1) 2000–2012 (2) 2011/2012 for different types of SMI diagnoses compared with people without SMI and by the most affluent and most deprived quintile (3) the 5-year period 2007/2008–2011/2012 for the combinations of comorbidities, for both people with and without SMI	Range of population from 2000–2012: 19,658–33,117	Prevalence of CKD amongst people with SMI increased from 0.28% in 2000/2001 to 8.24% in 2011/2012 The prevalence increased in those without SMI but not to the same extent, increasing from 0.26% in 2000/2001 to 4.16% in 2011 (although the prevalence decreased from 4.27% in 2009/2010)	The prevalence of CKD amongst people with SMI increased between 2000 to 2012, more than those without SMI, where the prevalence of CKD decreased from 2009–2012
Tonelli et al. [30]	Journal article	Alberta, Canada	To identify the associations between burden of comorbidity and adverse outcomes in people with CKD	Retrospective population-based cohort study	2003–2011	*N* = 530,771 Mean age = 56 Male = 42.4%	Patients with schizophrenia = 6227 (1.2%) Schizophrenia was associated with population attributable risk for hospitalisation Mental health conditions and chronic pain (including schizophrenia) associated with a substantial proportion of the burden of hospitalisation or death	Schizophrenia is associated with a higher risk of hospitalisation in people with CKD
Tzeng et al. [21]	Journal article	Taiwan, Taiwan’s National Health Insurance Database	To establish whether there is an association between schizophrenia and CKD	Nationwide matched cohort study	2003–2007	With schizophrenia *N* = 2338 Controls *N* = 7014 Patients with Schizophrenia Female = 47.9% Comorbidities: Hypertension = 17.96% Diabetes mellitus = 9.32% Heart disease = 14.70%	Incidence rate of CKD per 1000: With schizophrenia = 25.13 Without schizophrenia = 18.6 Crude HR: 1.36 (1.13 TO 1.63) CKD-free survival curves obtained by the Kaplan–Meier method, and shows that patients with schizophrenia had a significantly lower 3-year CKD-free survival than those without (log rank test *P* < 0.05)	People with schizophrenia were at a higher incidence of CKD and had a lower 3-year CKD free survival rate, compared to people without schizophrenia
McPherson et al. [35]	Journal article	USA, Washington state	To determine if co-occurring SMI independently increases the risk for rehospitalisation, particularly via the emergency department, among people across stages of CKD	Retrospective cohort study	April 2006–December 2008	Reference *N* = 548,532 CKD only *N* = 31,166 Median age = 75 SMI only *N* = 20,167 Median age = 53 CKD + SMI *N* = 717 Median age = 64	Rehospitalisation rates are significantly increased for both the CKD cohort and SMI cohort, but even greater for the CKD + SMI cohort (HR = 1.55, 95% ci = 1.40–1.73, *P* < 0.001) The risk of repeat hospitalisation resulting in death was greater in the CKD cohort, but not significantly greater in the SMI or CKD + SMI cohort	Co-occurring SMI independently increased the risk of rehospitalisation, particularly through the ED, for people with CKD. Thus, people with CKD and co-occurring SMI are at considerably increased risk for emergent rehospitalisations This risk was higher for those with CKD and co-occurring mood disorders (dysthymia, bipolar disorder, or depression), as opposed to schizophrenia. SMI comorbidities represent unique complications that should be further studied to mitigate risks of rehospitalisation in people with CKD. Addressing SMI could be an important strategy to reduce repeated emergent hospital admissions in this group of people
Dube et al. [42]	Conference abstract	United States, single-centre renal unit	To explore the outcomes of people who have bipolar disorder and kidney failure and received a kidney transplant	Retrospective cohort study	1/7/2002–31/12/2011	*N* = 15 Adult patients with kidney failure and bipolar disorder who have received a kidney transplant Mean age = 59.1 ± 8.1 Female = 66.7% White = 93.3% Live donor = 53.3% Pre-emptive transplant = 46.7% Time on dialysis (months) = 18.8 + 23.1 Time on waitlist (months) = 14.0 + 14.6	Patient survival = 80% 1 patient died of cardiac arrest 4 days after transplant 1 patient died of lymphoma at 5 months 1 patient died of unknown causes at 4 years Death censored graft survival = 92.3% Grafts lost to primary non-function = 1 Post-transplant psychiatric hospitalisation = 2 patients 1 patient required re-commencement on lithium therapy Acute rejection = 6.7%	Renal transplant can be performed safely in patients who have kidney failure and well-controlled bipolar disorder There is a risk of deterioration in mental health following renal transplant Well-controlled bipolar disorder should not be considered a contraindication to kidney transplant
Lally et al. [22]	Conference abstract	Community mental health teams–geographical location not reported (Likely London)	To assess the prevalence of CKD in a cohort of community-based patients with established psychotic illnesses	Cross sectional survey	Not reported	*N* = 313 Male = 184 Age = 43.27 ± 10.45 years Mean eGFR = 81.58 ± 10.51 mL/min	Prevalence of CKD defined as eGFR < 60 mL/min = 2.9% By stage: Stage 1 CKD = 46.6% Stage 2 CKD = 50.5% Stage 3 = 2.9% Stage 4 or 5 = 0%	The rate of Stage 3 CKD in this population is higher than that in the general population. This is not surprising considering the prevalence of metabolic syndrome in this cohort It is likely that this population is at an increased risk of higher rates of CKD in the future due to their relatively young age
Smith et al. [23]	Journal article	Scotland, UK, Primary Care Clinical Informatics Unit	To examine the nature and extent of physical comorbidities in individuals with bipolar disorder within primary care, and assess whether they experience inequitable prescribing for CHD and hypertension	Cross sectional analysis	31^st^ March 2007	*N* = 2,582 (with bipolar disorder) 39.5% Male Mean age: 54.5 years	189 patients with bipolar disorder had a diagnosis of CKD OR: 2.42 (95% CI = 2.04 to 2.86 (P < 0.001))	People with bipolar disorder had higher rates of CKD (7.3% in bipolar disorder vs 2.4% in controls)
Welsh et al. [24]	Journal article	USA, Houston Outreach, Medicine, Education and Social Services (HOMES) Clinic	To assess the prevalence of bipolar disorder and schizophrenia, comparing demographics, health insurance status, comorbid medical conditions, and social habit data of patients with these patients	Retrospective healthcare records analysis (cross-sectional)	May 2007–May 2008	286 patients in total: 25 people had a diagnosis of schizophrenia (8.7%) 45 people had a diagnosis of bipolar disorder (15.7%)	People with schizophrenia and bipolar disorder were more likely to have certain comorbid conditions, including kidney disease (10% of patients with schizophrenia and bipolar disorder had kidney disease, in comparison to 2% of those without) (*P* =0.01)	Kidney disease is significantly more common in people with schizophrenia and bipolar disorder compared to those without
Bayat et al. [43]	Journal article	Lorraine, France–NEPHROLOR regional database	To describe medical and non-medical factors associated with registration on the transplant waiting list, and to depict factors linked to registration before start of dialysis	Retrospective longitudinal cohort study	1/7/1997–30/6/2003	All adult patients living in Lorraine and starting KRT (dialysis and pre-emptive transplantation) 1725 adults started KRT Mean age: 65.4 ± 15.3 Male: 57.9% Diabetes: 33.2% Cardiovascular disease: 38% Registered for transplant: 24.7% of the patients under 80 were registered for transplant waiting list Registered before first KRT: 18.7%	Psychiatric disorders are an independent factor associated with non-registration Psychiatric disorder: Registered: 5 (1.4%) Nonregistered: 60 (5.3%) *P* = 0.001	The presence of a psychiatric disorder strongly and independently limited access to the waiting list
Abbott et al. [33]	Journal article	United States, United States Renal Data System	To determine the rate of hospitalisations for a primary and secondary diagnosis of psychoses in renal transplant recipients in comparison with patients with end-stage renal disease on chronic dialysis, and its relationship to clinical outcomes and mortality	Retrospective cohort study	July 1994–May 2000	Renal transplant recipients *N* = 42,096 Mean age = 43.4 ± 14.9 Male = 60.1% African American = 23.1% Cadaveric donor = 69.1% RTR with an episode of psychosis *N* = 420 Mean age = 44.1 ± 14.5 Male = 58.8% African American = 24.5% Cadaveric donor = 79.5%%	Unadjusted HR for 1° psychoses: Graft loss = 2.76 (2.24–3.40) Rejection = 1.98 (1.61–2.43) Adjusted HR for 1° psychoses Graft loss = 2.97 (2.19–4.02) Rejection = 1.36 (1.06–1.74) Most frequent SMI primary and secondary diagnoses for RTR hospitalised psychoses (n (%)): Primary diagnoses Psychotic depression = 204 (49%) Schizophrenia = 6 (1%) Secondary diagnoses Psychotic depression = 43 (36%) Schizophrenia = 17 (8%) Cumulative mortality after hospitalised psychoses was 53% at 1 yr, 73% at 2 yr, and 84% at 3 yr. Hospitalised psychoses were also independently associated with increased risk of mortality in chronic dialysis patients as a time-dependent variable (AHR for mortality, 1.87; 95% CI, 1.77–1.98; *P* < 0.001, in Cox regression analysis adjusted for all factors	Hospitalisation for psychosis is associated with poor survival outcomes amongst both renal transplant recipients and people receiving haemodialysis Hospitalisation for psychosis is associated with an increased risk of graft loss and rejection for RTR Psychosis does not appear to be more common following RT, compared to amongst people receiving haemodialysis The underlying cause of psychosis is not associated with clinical outcomes or mortality in either RTR or haemodialysis patients

### Risk of developing CKD amongst people with SMI

Several studies examined the risk of developing CKD for people with SMI [2, 17–24]. There appears to be an increased risk for developing CKD amongst people with SMI [24], however the degree of risk may be different across SMI diagnoses. Tzur Bitan et al. [17] conducted a retrospective cohort study, comparing the prevalence of CKD amongst people with schizophrenia to matched controls without schizophrenia, over a 7-year period. Schizophrenia was found to be predictive of CKD above all other risk factors (OR = 1.62, 95% CI = 1.45–1.82, *P* < 0.0001) [17]. While other studies also found an increased risk of CKD amongst people with schizophrenia [21], the risk of CKD appeared to be higher for people with bipolar disorder. Smith et al. (2013) conducted a cross-sectional study exploring the prevalence of medical comorbidities in people with a diagnosis of bipolar disorder [21]. People with bipolar disorder had higher rates of CKD compared to controls (OR: 2.42, 95% CI = 2.04–2.86, *P* < 0.001). However, no studies carried out a direct comparison between SMI diagnoses.

The prevalence of CKD amongst people with SMI is increasing over time, according to a retrospective cohort study evaluating the prevalence of long-term conditions amongst people with SMI across a 12-year period [20]. The prevalence of CKD amongst people with SMI increased from 0.28% in 2000/2001 to 8.24% in 2011/2012. Whilst the prevalence of CKD also increased in people without SMI (from 0.26% in 2000/2001 to 4.16% in 2011/2012), the increase was at a slower rate, with the prevalence remaining lower in comparison.

### Risk factors for developing CKD amongst people with SMI

Three studies explored the role psychiatric medications play in the development of CKD, specifically first- and second-generation antipsychotics [25, 26], and anticonvulsants used as mood stabilisers in bipolar disorder [27].

Second-generation antipsychotics were associated with a small to moderate increase in risk of incident CKD in a case–control cohort study[25]. This study included 21,434 CKD cases and examined the association between second-generation antipsychotic use and incident CKD. They found an increased risk of CKD both in people who were using second-generation antipsychotics at the time of the study (OR 1.26, 95% CI: 1.12–1.42), and people who had ever used second-generation antipsychotics (OR 1.24, 95% CI:1.12–1.37). There did not appear to be a dose–response relationship, as both short- and long-term prescriptions were associated with an increased risk. All second-generation antipsychotics, except for aripiprazole, were associated with an increased risk of CKD, with the highest risk associated with clozapine [OR 1.81, 95% CI: 1.22–2.69]. In contrast, a study by Wang et al. [26] found there was no significant association between first- or second-generation antipsychotic use and CKD, but there was a significantly increased risk of CKD amongst people who used a combination of first- and second-generation antipsychotics (OR 1.28, 95% CI = 1.11–1.47). However, more than 85% of their sample were receiving a combination treatment of first- and second-generation antipsychotics, limiting their ability to draw definitive conclusions about the relative risk of different antipsychotics.

The role of anticonvulsants in the risk for CKD was explored by Kessing et al. [27] who compared the rates of CKD and kidney failure among people who had bipolar disorder who were treated with lithium, anticonvulsants, and people without bipolar disorder who had never been exposed to these drugs. Anticonvulsants and other mood stabilisers were associated with an increased risk of CKD. There was a dose–response relationship with anticonvulsant treatment, as people who received over 60 prescriptions of anticonvulsants were at a higher risk of developing CKD (HR = 2.30 [95% CI, 1.53–3.44]) compared to people who had only 1–2 prescriptions (HR = 1.23 [95% CI, 0.76–1.99]; *P* < 0.001). A similar pattern was also observed with kidney failure, as the rates of kidney failure increased with the number of anticonvulsant prescriptions a person had received (*P* = 0.002). This was in comparison to lithium, which did not have a significant association with the development of kidney failure.

### Outcomes for people with SMI and co-existing CKD

#### Suicide

One study explored suicide in the context of physical multi-morbidity and SMI. Chen et al. [28] explored the presence of physical comorbidities in the 3-month period before suicide mortality in individuals with bipolar disorder. People were more likely to have a higher comorbidity score and were more likely to have had a diagnosis of CKD in the 3-month period before they died by suicide.

#### Mortality

Several studies highlighted the increased risk of mortality amongst people who have co-existing SMI and kidney failure [29, 30]. Over a 5-year period people with schizophrenia have a lower risk of commencing dialysis, but a significantly higher risk of death, compared to people without schizophrenia [31]. Kimmel et al. [32] examined the association between hospitalisations with psychiatric diagnoses and death in adult patients receiving dialysis. There was an increased risk of 1-year mortality for people with kidney failure who had been hospitalised with a primary (OR 1.23, 95% CI: 1.18–1.28) or secondary psychiatric diagnosis (OR 1.09, 95% CI: 1.08–1.11), compared to those hospitalised without a psychiatric diagnosis. A study by Abbott et al. [33] found that the cumulative mortality following hospitalisation for psychosis for people who have co-existing kidney failure was 53% at 1-year follow-up, 73% at 2-year follow-up, and 84% at 3-year follow-up, and hospitalisation for psychoses was independently associated with mortality risk (HR = 1.87, 95% CI: 1.77–1.98; *P* < 0.001).

One study explored risk factors for mortality in people who had schizophrenia and kidney failure [34]. There was a significantly increased risk of mortality amongst people who had co-existing schizophrenia and kidney failure if they were over the age of 35, used a central venous catheter for haemodialysis access, and had heart failure. These are also associated with an increased risk of mortality in people without co-existing SMI. However, there was a reduced risk of mortality amongst people with co-existing schizophrenia and kidney failure if they had hypertension, a history of suicidal ideation or drug dependence, had ischaemic cardiovascular disease or a history of being non-compliant with treatment. These factors are associated with an escalation in care, close monitoring, and regular follow-up, which was thought to account for the reduction [34].

Most research available exploring mortality for people with co-existing SMI and CKD focused on kidney failure, as opposed to earlier stages of CKD. This may under-estimate the role that CKD plays in mortality for people with co-existing SMI, as people with SMI may not progress to kidney failure as they die before reaching the final stages [31].

#### Hospitalisation

McPherson et al. [35] explored the relationship between co-existing SMI and CKD, and rates of hospitalisation and repeated hospitalisation. Rehospitalisation rates are significantly increased both in people who have CKD, and people who have SMI, however there is a higher rate amongst people who have co-existing SMI and CKD (HR = 1.55, 95% CI = 1.40–1.73, *P* < 0.001), particularly those admitted through the emergency department. In contrast, while there was not a significantly increased risk of repeated hospitalisation for people with co-existing SMI and CKD, there was a significantly increased risk of emergent rehospitalisation. This risk was higher for those with affective disorders, such as bipolar, compared to those with schizophrenia [35, 36].

#### Outcomes for transplantation

Several studies that assessed clinical outcomes focussed on transplantation [37–42]. Three studies compared outcomes following kidney transplantation in people who have bipolar disorder to those who did not have bipolar disorder [37–39]. They found no significant differences in patient survival [37–39], graft survival [37–39], and rejection episodes [38, 39]. These similar outcomes were also seen in studies that compared transplant outcomes for people who had other SMI diagnoses, including schizophrenia [41], and psychosis [40].

None of the included studies found an increased risk of poor clinical outcomes for people who had a diagnosis of SMI and received a kidney transplantation, although there may be a risk of exacerbation of SMI symptoms in the immediate post-transplant period. Kofman et al. [39] found that following transplantation, 23 patients from their sample had a relapse of their psychiatric disorder[39], while 13 people required psychiatric hospitalisation.

### Access to specialist nephrology care

Despite the increased prevalence of CKD amongst people with SMI, there is evidence that people with SMI had lower rates of accessing specialist nephrologist care [17, 31]. Hsu et al. [31] found that people with schizophrenia and co-existing kidney failure had a lower chance of receiving an appointment with a nephrologist (OR = 0.6, 95% CI 0.4–0.8, *P* < 0.001), of receiving an erythropoietin prescription (OR = 0.7, 95% CI 0.6–0.9, *P* < 0.05), and were more likely to be hospitalised within the first year of commencing dialysis (OR = 1.4, 95% CI 1.0–1.8, *P* < 0.05).

Most studies exploring access to nephrology care focused on access to kidney replacement therapy. People with SMI and kidney failure were less likely to have received a kidney transplant (OR = 5.43, 95% CI = 2.84–10.38, *P* < 0.001) [17, 19], be on the register to receive a kidney transplant (only 1.4% of people registered for a kidney transplant had a psychiatric disorder, compared to 5.3% of people who were not registered) [43] or be scheduled for a kidney transplant evaluation appointment (only 6% patients with schizophrenia, who met the eligibility criteria for a kidney transplant, were scheduled for a transplant evaluation appointment) [44] compared to people without SMI. While people with SMI were more likely to commence haemodialysis than to receive a kidney transplant [19], there is evidence that in general they are less likely to commence kidney replacement therapy [17, 31]. Tzur Bitan et al. [17] conducted a retrospective cohort study and found dialysis treatment was more commonly received among patients with CKD who did not have a comorbid schizophrenia diagnosis (11.1%), compared to CKD patients who did have the diagnosis (8.5%).

### Barriers and facilitators of care

One study explored the experiences of renal nurses providing care to people with SMI receiving haemodialysis [45]. The main barriers to providing effective care were staff shortages, and a lack of staff education on mental illness. Nurses at times perceived people with SMI as difficult and challenging, particularly when they displayed aggressive behaviour. However, some facilitators included having effective communication skills and empathy. Renal nurses reported that they wanted more support from mental health teams, with a collaborative approach to patient care between teams.

## Discussion

This scoping review is the first to identify and describe the available evidence on the relationship between CKD and SMI. While several studies provided insight into the risk of CKD for people with SMI, contributing factors, clinical outcomes, and access to care, overall, there is a dearth of high-quality research focussed on co-existing SMI and CKD.

The evidence demonstrates there may be a significantly increased risk of CKD for people who have SMI [17–21, 31]. The level of risk appears to differ depending on the SMI diagnosis [19, 21, 27], although there is little research available examining the different prevalence rates across diagnoses. Additionally, there are few studies examining the impact of risk factors for CKD amongst people who have SMI. People who have SMI are more likely to have co-morbid health conditions such as type 2 diabetes and cardiovascular disease [2], are more likely to smoke [46], have metabolic syndrome [4], and live a sedentary lifestyle [47]. These factors all contribute to the risk of CKD, however none of the studies identified in this review examined the role of these risk factors in the development of CKD amongst people with SMI.

An interesting finding from the review was the relationship between psychiatric medication, other than lithium, and the risk of CKD. Two studies implicated both first- and second-generation antipsychotics in the risk of CKD [25, 26], with mixed findings, and clozapine was associated with the highest risk. Clozapine is a highly effective atypical antipsychotic with an extensive side effect profile and, like most antipsychotic medication [9], is associated with the development of metabolic syndrome, type 2 diabetes, and cardiovascular disease [48]. Yet there is limited research acknowledging the potential impact of medication for SMI on kidney health, with the exception of lithium treatment [16].

This review also highlights potential inequality in access to specialist care for CKD among people with SMI, particularly kidney transplantation. People who have SMI appear to have similar outcomes following transplantation, with no evidence of increased rejection rates, graft loss, or mortality [38, 39, 41]. Despite this, people with SMI were less likely to receive a kidney transplant, be on the transplant register, or be assessed for transplantation [17, 43]. This inequality in access to transplantation services for people with psychiatric diagnoses has been highlighted across specialties [49]. This lack of access could result from stigma towards people with SMI, a lack of knowledge of the guidelines and evidence, and therapeutic pessimism [50]. Further research is needed to explore the role of stigma as a barrier to transplant services for those with SMI, in order to address this inequality [49].

This inequality is compounded by worse clinical outcomes overall. The evidence highlights higher rates of mortality and hospitalisation for people who have CKD and SMI [31, 33]. While people who have SMI are less likely to reach kidney failure, this is most likely a result of them dying too early for their disease to progress [31]. However, while people with SMI are not reaching the end stages of CKD, this does not mean that CKD does not contribute to the mortality gap. Cardiovascular mortality is a significant contributor to the mortality gap for people with SMI [51], while people with CKD are at a significantly raised risk of cardiovascular mortality, even in the earlier stages of the disease [30, 52].

There are a few strengths and limitations to this review. Articles were restricted to those published in the English language, meaning there may be relevant articles published in other languages that would have contributed to our understanding of the relationship between CKD and SMI, but were not accessed. Studies were included on the basis that they reported information related to people who had been diagnosed with a mental health condition classed as an SMI, however the reliability of diagnosis was not assessed. Additionally, since this is a scoping review the quality of the studies was not formally assessed using a risk of bias tool as the aim of the review was to describe the evidence, as opposed to evaluating the reliability of results. However, this review is the first to provide an overview of the literature available on the relationship between CKD and SMI. Moreover, using the scoping review methodology enabled a broad overview of the evidence to identify key gaps in the research. Table 4 illustrates some of these gaps in the evidence.

**Table 4:** Key gaps in evidence

*The epidemiology of CKD and SMI*
• What is the true prevalence and incidence of CKD amongst people with SMI, across all stages of the disease? • What is the role of risk factors in the development of CKD amongst people with SMI? • What is the role of risk factors in the progression of CKD and associated complications of CKD for people who have SMI? • How does CKD contribute to the mortality gap? • How does CKD contribute to mental health outcomes?
*The management of CKD amongst people who have SMI*
• How is CKD monitored and managed in primary care and secondary care services for people who have SMI? • How does the monitoring and management of CKD differ from other long-term conditions, such as type 2 diabetes and cardiovascular disease, for people who have SMI? • What is the experience of CKD and/or kidney failure for people who have co-existing SMI? • What are the barriers and facilitators to access to nephrology care for people who have SMI? • What does optimal nephrology care look like for people who have SMI?
*The management of kidney failure amongst people who have SMI*
• How does kidney failure impact SMI, in terms of symptoms and treatment? • What does optimal dialysis care look like for people who have SMI? • What are the support needs of people who have SMI and kidney failure? • What are the support needs of healthcare professionals providing care to people with SMI and kidney failure? • Why are people with SMI less likely to receive a kidney transplant? • What are the barriers and facilitators to kidney transplantation for people who have SMI?

In conclusion, there is a dearth of research exploring the prevalence, risk factors, outcomes, and available care for people with SMI and CKD. There was a significant gap in relation to qualitative research, particularly studies exploring the associated care pathways for people with SMI and CKD, how this is monitored and managed in primary or secondary care mental health services, and how this compares to other long-term conditions associated with SMI, such as diabetes, cardiovascular disease and chronic obstructive pulmonary disease. The available evidence suggests there is an increased risk of CKD amongst people who have SMI, that people with SMI have poorer clinical outcomes from CKD, and that they are not accessing specialist renal care to the same degree as people without SMI. There is a need for further research in this area to fully understand this relationship and inform strategies to improve clinical outcomes and close the mortality gap.

## Data Availability

No new data were generated as part of this review.
